# Immunotherapy in Gastric Cancer

**DOI:** 10.3390/curroncol29030131

**Published:** 2022-03-02

**Authors:** Anica Högner, Markus Moehler

**Affiliations:** 1 Campus Virchow-Klinikum, Medizinische Klinik m.S. Hämatologie, Onkologie und Tumorimmunologie, Charité-Universitätsmedizin Berlin, Corporate Member of Freie Universität Berlin, Humboldt-Universität zu Berlin and Berlin Institute of Health, Augustenburger Platz 1, 13353 Berlin, Germany; anica.hoegner@charite.de; 2 Universitätsmedizin der Johannes Gutenberg-Universität Mainz, 55131 Mainz, Germany

**Keywords:** checkpoint inhibition, CTLA-4, esophagogastric cancer, Her2-positive, immunotherapy, gastric cancer, PD-L1, PD-1, trastuzumab

## Abstract

Immune checkpoint inhibition is a new standard of targeted therapy in the treatment of advanced or metastatic gastric cancer (GC) and is represented in various combinations with and without chemotherapy in every therapy line within clinical trials. In advanced adenocarcinoma of GC, gastroesophageal junction cancer (GEJC) and esophageal cancer (EC), the combination of nivolumab and chemotherapy in first-line therapy improves overall survival (OS) in PD-L1 (programmed cell death protein 1)-positive patients with approval in Europe (PD-L1 CPS (combined positivity score) ≥ 5), USA and Taiwan (CHECKMATE-649) and pembrolizumab plus chemotherapy for GEJC and EC in Europe (CPS ≥ 10) and the USA (KEYNOTE-590). Furthermore, pembrolizumab plus trastuzumab and chemotherapy show clear benefits in OS and are approved as first-line treatment of Her2 (human epidermal growth factor receptor-2)-positive tumors in the USA (KEYNOTE-811). Nivolumab demonstrates superior OS regardless of PD-L1 expression in third-line therapy with approval in Japan (ATTRACTION-02) and pembrolizumab prolonged the duration of response in PD-L1 positive patients with approval in the USA in PD-L1 CPS ≥ 1 patients (KEYNOTE-059). This review reflects the rationale and current results of phase II and III clinical trials investigating various immune checkpoint inhibitors targeting PD-L1/1 and CTLA (anticytotoxic T-lymphocyte-associated antigen)-4 in combination with and without chemotherapy and Her2-targeted therapy in GC.

## 1. Introduction

The global incidence rate of GC accounts for approximately 1,089,103 cases, ranking gastric cancer in sixth place among newly diagnosed cancer cases worldwide. With approximately 769,000 deaths due to GC per year, it constitutes the third most common cause of death from cancer [1]. In Europe, approximately 133,100 GC cases are newly diagnosed and approximately 102,200 patients die from GC, which ranks GC as the fifth most common cancer in European men and sixth most common in women [2]. Currently, the 5-year survival rate is 32% [3]. With over 50% of patients being diagnosed after the cancer has already spread, GC requires an effective and feasible therapy in an individual multimodal setting.

The established combination chemotherapy of esophagogastric adenocarcinomas (EGC) has lately been enriched by the rapidly developing field of immune checkpoint inhibition. On the basis of phase II and phase III trials, the PD-1 (programmed death 1) inhibitors nivolumab and pembrolizumab were already approved in mono- and in combination therapy of advanced GC in first- or third-line settings in Europe, the USA and Taiwan (see Table 1).

The diversity of immune checkpoint inhibitors to different molecular targets on tumor cells and immune cells is growing and is currently being investigated in several clinical trials in GC: the anti-PD-1 antibodies nivolumab, pembrolizumab, sintilimab, tislelizumab, retifanlimab and tebotelimab (T-cells), as well as the anti-PD-L1 antibodies atezolizumab, avelumab, durvalumab, retifanlimab, tebotelimab (cancer cells, dendritic cells) and the anti-CTLA-4 antibody ipilimumab (T-cells). In particular, the combination of chemo- and immunotherapy with a possible synergistic effect of T-cell recruitment and activation is the object of current research.

An already established therapeutic approach in targeted therapy of progressive GC is the Her2-blockade in patients with Her2-positive tumors with a rate of 15–20% Her2 overexpression [4]. Here, trastuzumab has been successfully implemented as standard of care in combination with chemotherapy in the first-line setting of patients with advanced GC [5]. In the new era of immune checkpoint inhibition, combination therapies of Her2- and PD-1/PD-L1-directed therapies aim for synergistic effects. Several global phase II and III trials analyze the addition of pembrolizumab, nivolumab and ipilimumab to standard of care first-line therapy of trastuzumab plus chemotherapy in Her2-positive advanced cancer patients [6].

As there remain Her2-positive patients who do not benefit from trastuzumab, various innovative approaches of Her2-directed therapy are under investigation, including the promising antibody-drug conjugate trastuzumab-deruxtecan (T-DXd) in patients who were progressive under prior trastuzumab therapy [7]. T-DXd has already been approved by the FDA as a second Her2-directed therapy option for patients with unresectable, locally advanced or metastatic GC in 2021 and is currently under investigation as a combination therapy with immune checkpoint inhibitors [8,9]. Another approach is the use of the Fc-engineered Her2-directed monoclonal antibody margetuximab. This has already shown positive effects on overall response and disease control in combination with PD-1 inhibition with pembrolizumab in a phase I/IIb clinical trial and is being further tested with retifanlimab or tebotelimab in a phase II/III trial [10,11].

The number of GC patients with progress after first- and second-line therapy grows and emphasizes the role of third- and last-line treatment options. Results of a systematic review and meta-analysis of seven randomized controlled trials indicate a superiority in efficacy of third- and later-line therapies in advanced GC patients [12]. Although more toxicities occurred in third-line treatment regimens, the overall safety profile remains manageable and encourages the use of immunotherapy in single or combination application in later therapy lines.

This article provides an overview of phase II and III clinical trials investigating the role of immune checkpoint inhibition as targeted therapy in addition to standard chemotherapy regimens +/− Her2-targeted therapy in various therapy lines and reflects their impact on the current treatment regimens and actual approvals for immune checkpoint inhibitors in EGC (Table 1).

**Table 1:** Overview of clinical trials of immune checkpoint inhibitors in esophagogastric adenocarcinoma.

Therapy Line	Agent	Target Structure	Trial	Author	Reference	Phase	Study Design	Approval Europe	Approval USA	Approval Japan	Approval Taiwan
Perioperative	Atezolizumab	PD-L1	Dante	Al-Batran et al.	[13]	II	Atezolizumab + FLOT vs. FLOT perioperative				
	Nivolumab, Ipilimumab	PD-1/CTLA-4	Vestige	Smyth et al.	[14]	II	Nivo+Ipi vs. chemo continuation				
	Pembrolizumab	PD-1	KN-585	Bang et al.	[15]	III	Pembro vs. placebo + chemo (cis+cape/5-FU, FLOT)				
	Durvalumab	PD-L1	Matterhorn	Janjigian et al.	[16]	III	Durvalumab vs. placebo + FLOT				
First-line	Nivolumab	PD-1	CM-649	Janjigian et al.	[17]	III	(Nivo/Ipi) vs. FP vs. FP + nivo	yes (CPS ≥ 5)	yes		yes
	Nivolumab	PD-1	Attraction-04	Boku et al.	[18]	II/III	Nivo vs. placebo + chemo (SOX/CAPOX)				
	Pembrolizumab	PD-1	KN-590	Sun et al.	[19]	III	Pembro vs. placebo + FP	yes (CPS ≥ 10)	yes		
	Pembrolizumab	PD-1	KN-062	Shitara et al.	[20]	III	Pembro vs. pembro + chemo vs. chemo (cis/5-FU, cape)				
	Pembrolizumab	PD-1	KN-859	Tabernero et al.	[21]	III	Pembro vs. placebo + cis + FP/CAPOX				
	Avelumab	PD-L1	Javelin Gastric 100	Moehler et al.	[22]	III	Avelumab maintenance				
	Sintilimab	PD-1	Orient-16	Xu et al.	[23]	III	Sintilimab vs. placebo + chemo (XELOX)				
	Tislelizumab	PD-1	Beigene-305	Xu et al.	[24]	III	Tislelizumab vs. placebo + chemo (oxali+cape/cis+5-FU)				
Second-line	Pembro	PD-1	KN-061	Shitara et al.	[25]	III	Pembro mono vs. chemo (paclitaxel)				
	Avelumab	PD-L1	RAP	Högner et al.	[26]	II	Avelumab + ramucirumab + paclitaxel				
Third-line	Nivolumab	PD-1	Attraction-02	Kang et al.	[27]	III	Nivo vs. placebo			yes	
	Pembrolizumab	PD-1	KN-059	Fuchs et al.	[28]	II	Pembro mono		yes (CPS ≥ 1)		
	Avelumab	PD-L1	Javelin Gastric 300	Bang et al.	[29]	III	Avelumab vs. chemo (physician’s choice)				
Her2 pos cancer											
Perioperative	Pembro + Tmab	PD-1/Her2	Pherflot	in process	-	II	Pembro + Tmab + FLOT *(in process)*				
First-line	Pembro + Tmab	PD-1/Her2	KN-811	Janjigian et al.	[6]	III	Pembro vs. placebo + Tmab + FP	expected in 2023	yes		
	Nivo + Ipi + Tmab	PD-1/CTLA-4/Her2	Intega	Stein et al.	[30]	II	Tmab + Nivo + Ipi vs. FOLFOX + Tmab + Nivo				
	Durvalumab + T-DXd	PD-L1/Her2	Destiny-Gastric 03	Janjigian et al.	[31]	Ib/II	T-DXd +/− Durvalumab +/− Chemo				
	Retifanlimab + Tebotelimab + Margetuximab	PD-1/PD-1/Her2	Mahogany	Catenacci et al.	[11]	II/III	Margetuximab, Retifanlimab, Tebotelimab +/− Chemo				

Pembro = pembrolizumab, Nivo = nivolumab, Tmab = trastuzumab, T-DXd = trastuzumab-deruxtecan, PD-1 (programmed death-ligand 1), PD-L1 (programmed cell death protein 1), CPS (combined positivity score), chemo = chemotherapy, cis = Cisplatin, cape = Capecitabine, FP = Fluoropyrimidine.

## 2. Curative/Perioperative Therapy

In total, 30–40% of the patients with adenocarcinoma of the stomach are candidates for potentially curative surgery at the time of diagnosis. The positive effect of implementation of checkpoint inhibition in the perioperative setting is under investigation in several clinical trials.

The selective anti-PD-L1 antibody atezolizumab was investigated in the randomized phase II DANTE trial of the AIO (Arbeitsgemeinschaft Internistische Onkologie) in patients with resectable, localized EGC [32]. Patients were randomized 1:1 in the experimental combination arm with atezolizumab plus chemotherapy (FLOT: 5-fluorouracil, folinic acid, oxaliplatin, docetaxel) and the standard arm of mono-chemotherapy. Primary endpoints were progression-free survival (PFS) and disease-free survival (DFS). The first results of the safety analysis demonstrate a feasible and safe application of atezolizumab in combination with FLOT in the perioperative setting and first efficacy results are soon to be expected [13].

In the VESTIGE trial, a phase II trial of the EORTC (European Organisation for Research and Treatment of Cancer), adjuvant immunotherapy in patients with locally advanced EGC is analyzed after neoadjuvant chemotherapy with FLOT followed by surgery. Inclusion criteria comprise the selective inclusion of patients with a high risk of recurrence after surgery (ypN+ and/or R1 resection) [14]. After neoadjuvant chemotherapy, patients will be randomized in a 1:1 ratio into the study arms with either continuation of neoadjuvant chemotherapy or double checkpoint blockade with nivolumab and ipilimumab. The study will recruit 240 patients. The primary endpoint is an improvement in DFS.

The administration of pembrolizumab in the perioperative setting of EGC is evaluated in the KEYNOTE-585 trial, a global double-blind phase III trial with an initial planned enrollment of 860 patients. Patients will receive postoperative pembrolizumab plus chemotherapy (cisplatin + capecitabine/5-FU) versus placebo plus chemotherapy in a 1:1 randomization ratio. Based on the results of the FLOT4 trial [33], the FLOT chemotherapy backbone was also included in the Keynote-585 trial [15]. 

A benefit to OS of the PD-L1 inhibitor durvalumab is investigated in the perioperative setting independent of PD-L1 expression status in combination with FLOT versus FLOT alone within the MATTERHORN trial, a global double blind placebo controlled phase III trial with a recruitment plan of 900 patients with resectable EGC [16]. The primary endpoint is event-free survival (EFS) evaluated by blinded independent central radiology and/or local pathology testing.

Currently, checkpoint inhibitors in the perioperative setting of GC are only available within clinical trials.

## 3. Palliative First-Line Therapy

We and others presented the substantial results of the CHECKMATE-649 trial, a global phase III trial, at the ESMO 2020, ASCO 2021 and ASCO-GI 2022. The trial investigated the effect of a chemotherapy-free combination of nivolumab plus ipilimumab versus nivolumab plus chemotherapy (FOLFOX/XELOX) versus chemotherapy alone in a three-armed trial design. The large patient population of 1581 patients (24% Asian, 76% Non-Asian, 100% adenocarcinoma) tested the chemo-containing regimens; 60% of these patients (*n* = 955) had a PD-L1 CPS score of ≥5. The combination of nivolumab plus chemotherapy achieved a significant benefit to OS for both the primary endpoint group with PD-L1 CPS ≥ 5 tumors (median OS 14.4 vs. 11.1 mths (HR 0.71 (98.4% CI (0.59–0.86)), *p* < 0.0001)) and the group of all patients (median OS 13.8 vs. 11.6 months (mths) (HR 0.80 (99.3% CI 0.68–0.94), *p* = 0.0002)). The surviving patients after 12 months with PD-L1 CPS ≥ 5 were meaningfully higher in the combination arm of nivolumab plus chemotherapy versus chemotherapy alone (57% vs. 46%). The nivo-chemotherapy combination therapy also improved PFS (HR 0.68 (98% CI 0.56–0.81), *p* < 0.0001) with a reduction in mortality rate of 32% [17]. Through all CPS subgroups, an improvement of overall response rates (ORR) for the nivo-chemotherapy combination was achieved. Patients with PD-L1 CPS ≥ 5 and MSI (microsatellite-instability)-high tumors especially profited from the combination with immunotherapy. The chemotherapy-free combination of nivolumab and ipilimumab showed no clear benefit in OS compared to chemotherapy alone. Based on these results, the FDA (Food and Drug Administration) and the TFDA (Taiwan Food and Drug Administration) approved nivolumab plus chemotherapy in patients with advanced/metastatic esophageal/GEJC/gastric cancer independent from PD-L1 CPS status in the USA and Taiwan, respectively. In Europe, the EMA (European Medicines Agency) approved nivolumab plus chemotherapy in patients with PD-L1 CPS ≥ 5 (Table 1). These results fortunately enable patients with advanced or metastatic GC to have access to a promising effective immune checkpoint inhibitor therapy in the first-line setting.

In the Asian ATTRACTION-04 trial [34], a multicenter phase II/III trial evaluated the combination of nivolumab plus chemotherapy (SOX or CapeOX) versus chemotherapy alone in patients with previously untreated advanced or recurrent EGC in first-line therapy. The combination of nivolumab and chemotherapy significantly improved median PFS (9.7 mths (5.8–not reached) and 10.6 mths (5.6–12.5)) [18]. A possible reason for the missing effect on OS in this trial (median OS > 17 mths in both arms) was probably the fact that many patients had received subsequent therapies and additional immunotherapy.

As presented at ESMO 2020, the KEYNOTE-590 trial showed a significant benefit of OS in 749 patients with locally advanced or metastasized squamous cell carcinoma of the esophagus (PEC, *n* = 73%) and adenocarcinoma of the gastroesophageal junction (*n* = 25%, Siewert type 1). In this randomized, double-blind phase III trial, patients received equally either pembrolizumab plus chemotherapy (cisplatin, 5-FU) versus chemotherapy alone. Independently from the CPS score and the tumor histology, the combination therapy with pembrolizumab showed a superior survival effect of OS (all patients 12.4 vs. 9.8 mths (HR 0.73 (95% CI 0.62–0.86), *p* < 0.0002) and PFS (all patients 6.3 vs. 5.9 mths (HR 0.65 (95% CI 0.55–0.76). The subgroup of squamous cell and adenocarcinoma patients with CPS ≥ 10 especially profited from the combination therapy (PEC: median OS 13.9 vs. 8.8 mths, HR 0.57 (95% CI 0.43–0.75); adenocarcinoma: median OS 12.1 vs. 10.7 mths, HR 0.83 (95% CI 0.52–1.34)). ORR was 45% in the combination of immune and chemotherapy (95% CI, 40–40) vs. 29% (95% CI, 25–34) in the chemotherapy [19]. Subsequently, the FDA approved pembrolizumab in combination with cisplatin and 5-FU independently from PD-L1 CPS scores (March 2021) and the EMA approved pembrolizumab for patients with CPS ≥ 10 (June 2021) for metastatic esophageal and gastroesophageal junction cancers.

Patients with PD-L1 positive (CPS ≥ 1) advanced EGC show a non-inferiority of pembrolizumab monotherapy compared to chemotherapy alone (cisplatin + 5-FU/capecitabine) in the final analysis of the phase III KEYNOTE-062 trial (10.6 vs. 11.1 mths, HR 0.91, 99.2% CI 0.69–1.18) [20]. Overall, 763 patients (69% gastric cancer) were randomized in three therapy arms, including pembrolizumab monotherapy, combination of pembrolizumab plus chemotherapy (cisplatin/5-FU or capecitabine) or chemotherapy plus placebo. While pembrolizumab mono was non-inferior versus chemotherapy in patients with PD-L1 CPS ≥ 1 (median OS 10.6 vs. 11.1 mths (HR 95% CI: 0.74 (0.74–1.10), *p* = 0.162), pembrolizumab monotherapy prolonged OS in patients with PD-L1 CPS ≥ 10 (median OS 17.4 vs. 10.8 mths, HR 0.69 (95% CI 0.49–0.97)). Up-to-date results from the additional 25 months of follow-up were presented at ASCO-GI 2022 and also confirm these findings [35]. However, crossing curves indicate that this subgroup comprises patients who seem to die faster with pembrolizumab therapy, whereas in contrast, another group of patients seem to live longer with pembrolizumab monotherapy. There was no difference in OS in pembrolizumab plus chemotherapy in both subgroups of patients with PD-L1 CPS ≥ 1 (OS 12.5 vs. 11.1 mths, HR 0.85 (95% CI 0.7–1.03, *p* = 0.05) and PD-L1 CPS ≥ 10 (OS 12.3 vs. 10.8 mths, HR 0.85 (95% CI 0.62–1.17), *p* = 0.16). In particular, the group of patients with PD-L1 CPS ≥ 1 and MSI-high tumors (*n* = 35) showed a benefit of pembrolizumab versus chemotherapy with prolongation of OS from 47% to 79% (HR 0.29, 95% CI) [20].

The effect of pembrolizumab vs. placebo plus chemotherapy on OS (primary endpoint) is further evaluated in the phase III KEYNOTE-859 trial with a planned 1542 patients with Her2-negative advanced unresectable or metastatic EGC. The aim of the study is to strengthen the evidence that combining pembrolizumab with standard-of-care chemotherapy improves survival of patients with advanced/metastatic cancer [21].

The effect of a maintenance therapy following first-line chemotherapy with avelumab, an additional PD-L1 inhibitor, was investigated in the phase III JAVELIN Gastric 100 trial. Avelumab maintenance was investigated in 499 patients with advanced EGC with stable disease after at least 12 weeks of first-line therapy. The primary objective of showing superior benefit in OS with avelumab maintenance was not met and duration of response could not be prolonged in this trial [22]. However, an exploratory subgroup analysis with the 22C3 antibody shows a good signal for using checkpoint inhibition for maintenance after effective induction of chemotherapy.

The Asian phase III ORIENT-16 trial in 650 patients with advanced adenocarcinoma of the stomach (61% with PD-L1 CPS ≥ 5) examined placebo-controlled PD-1 inhibitor sintilimab in combination with chemotherapy (XELOX). As presented at ESMO 2021, the first results show a survival benefit of the combination therapy in all randomized patients versus chemotherapy plus placebo (median OS 15.2 vs. 12.3 mths, HR 0.77 (95% CI 0.63–0.94), *p* = 0.0090). This effect was even more clear in the group of patients with PD-L1 CPS ≥ 5 tumors (median OS 18.4 vs. 12.9 mths (HR 0.66 (95% CI 0.51–0.86), *p* = 0.0023)) [23].

Tislelizumab, another previously reported well-tolerated immune checkpoint inhibitor targeting PD-1, is currently the subject of investigation in the BEIGENE-305 trial in patients with locally advanced unresectable or metastatic EGC. Approximately 997 patients are randomized 1:1 to receive tislelizumab or placebo in combination with chemotherapy (oxaliplatin plus capecitabine/cisplatin plus 5-FU). The primary endpoints of the study include PFS and OS [24].

In summary, for first-line therapy of adenocarcinoma of the stomach and gastroesophageal junction, the immune checkpoint inhibitor nivolumab in combination with chemotherapy (platin/fluoropyrimidine) was established as an approved feasible immune therapy in European patients with CPS ≥ 5, as well as pembrolizumab in combination with chemotherapy for EGC patients with CPS ≥ 10 (see Table 1). Furthermore, for patients with Her2-positive EGC, the FDA approved the combination of pembrolizumab, trastuzumab and chemotherapy in a first-line setting in the USA based on interim analysis of the KEYNOTE-811 trial [6], which is discussed in more detail in the anti-Her2 targeted therapy part of this review.

## 4. Palliative Second-Line Therapy

In the KEYNOTE-061 trial with pembrolizumab monotherapy versus paclitaxel chemotherapy, the primary endpoint (superior OS of PD-L1 CPS ≥ 1 patients) was not met in 592 patients with EGC and progress after first-line chemotherapy. However, the data show that the efficacy of pembrolizumab is dependent on the PD-L1 CPS score; patients with PD-L1 CPS ≥ 10 show a superior OS of pembrolizumab application in contrast to the group of patients with PD-L1 CPS ≥ 1 [25]. The role of tumor mutational burden (TMB) as a predictive marker of the response to immune checkpoint inhibition was discussed at the ASCO Annual Meeting 2020 and no correlation of TMB to PD-L1 CPS was found [36].

In addition, the PD-L1 inhibitor avelumab was investigated in patients with advanced EGC independently from PD-L1 CPS score in the single-arm phase II RAP trial. The aim of the study is to show a superior effect on survival with avelumab in combination with the standard second-line backbone of paclitaxel and ramucirumab (primary endpoint: OSR at 6 mths) [26]. The combination of immune checkpoint blockade with VEGF-targeted therapy plus chemotherapy is assumed to enhance the immunogenicity of tumor cells and, therefore, the response to immune checkpoint inhibition. The recruitment of the study is complete, and first results are expected soon.

## 5. Palliative Third-Line Therapy

The role of nivolumab in the therapy of advanced EGC after two therapy lines was evaluated in 493 patients independently from the PD-L1 status in the Asian phase III ATTRACTION-02 trial. Patients who received nivolumab versus placebo showed an ORR of 11.4% with improvement of OS (median OS 5.3 mths vs. 4.1 mths; HR 95% CI: 0.63 (0.51–0.78), *p* < 0.001, primary endpoint). After one year, 26.2% of patients treated with nivolumab were alive in contrast to 10.9% in the placebo group [27]. Based on these results, nivolumab was approved as monotherapy in Japan in 2017. In Europe, the approval was rejected due to the exclusive Asian patient population.

Pembrolizumab monotherapy was investigated independently from the PD-L1 expression status in the non-randomized phase II trial KEYNOTE-059 in patients who had progressive disease after at least two therapy lines. Pembrolizumab in particular showed a positive effect on ORR (primary endpoint) in PD-L1-positive cancer patients (PD-L1 CPS expression ≥ 1) as compared to PD-L1-negative patients (ORR 15.5% vs. 6.4%, median OS 5.8 vs. 4.6 mths). The duration of response (DOR) was also prolonged in the PD-L1-positive group (overall DOR 8.4 mths, PD-L1-pos: 16.3 mths) [28]. These data led to the approval of pembrolizumab monotherapy in the third-line setting for patients with progressive EGC with PD-L1 CPS ≥ 1 expression in the USA (see Table 1).

The JAVELIN Gastric 300 trial compared the administration of avelumab versus chemotherapy (physician’s choice) as a third-line treatment for patients with advanced EGC. In 371 randomized patients, the primary endpoint (improvement of OS: median OS 4.6 vs. 5.0 mths, HR = 1.1 (95% CI 0.9–1.4), *p* = 0.81) and the secondary endpoints of PFS and ORR were not met. Nevertheless, avelumab application was more safe compared to chemotherapy [29].

The application of checkpoint inhibitors (nivolumab/pembrolizumab, CPS ≥ 1) in the third-line therapy for European patients is currently recommended after a positive health insurance assessment.

## 6. Combination of Immune Checkpoint Inhibition and Her2-Targeted Therapy

Since the superior OS results of the TOGA trial with adding trastuzumab to first-line chemotherapy in patients with Her2-positive tumors (immunohistochemical expression level 3+ or 2+ combined with positive FISH-verification of HER2 gene amplification), trastuzumab was established as standard therapy in combination with chemotherapy in the first-line setting for Her2-positive patients with advanced or metastatic disease. Trastuzumab was also implemented in international treatment recommendations including ESMO guidelines [37]. In 446 Her2-positive patients (12%), a combination of Her2 blockade and chemotherapy (cisplatin + 5-FU/capecitabine) was superior with improvement in median OS by 4.2 months (HR = 0.65, 95% CI 0.52–0.83) [5]. Currently, the addition of trastuzumab to immune- and chemotherapy is investigated comprehensively in perioperative and palliative therapy settings.

In the perioperative setting, a single-arm phase II trial of the AIO study group PHERFLOT is being planned in patients with Her2-positive localized EGC. The aim is to demonstrate an improvement in DFS and an increase in the pathological complete response (pCR) rate upon combining Her2-blockade, PD-L1 inhibition and standard chemotherapy. The trial is currently in progress and recruitment of 30 patients starts this year. The rationale for this trial is, among others, based on the excellent efficacy of increasing the pCR rate and DFS by combining Her2- and PD-1-antibodies in the perioperative therapy setting (HERFLOT trial) [38].

In patients with advanced EGC, the combination of targeted therapy with trastuzumab, cytotoxic chemotherapy (oxaliplatin/cisplatin plus capecitabine/5-FU) and immune checkpoint inhibition (pembrolizumab) was initially investigated in a single-arm phase II trial in 37 patients with Her2-positive metastatic EGC (30% gastric cancer). The primary endpoint, PFS after 6 months, was reached in 70% of patients (*n* = 26/37, 95% CI 54–83). After 12 months, the majority of patients were still alive (OSR 80%, 95% CI 68–95). The duration of treatment was 10 months (IQR 5.7–13.7) [39]. 

The demonstrated safe and feasible administration of trastuzumab plus immune- and chemotherapy in the first-line setting with promising efficacy in Her2-positive metastatic EGC of this single-arm trial is further investigated in the randomized, double-blind phase III trial KEYNOTE-811. This trial evaluates the effect of pembrolizumab versus placebo in combination with trastuzumab and chemotherapy (5-FU + cisplatin/CAPOX: capecitabine and oxaliplatin) on OS and tolerability in an estimated 692 patients with Her2-positive metastatic EGC. The first interim analysis (IA1) of the first 264 patients showed superior ORR of 22.7% in the combination arm of pembrolizumab, trastuzumab and chemotherapy versus placebo plus trastuzumab and chemotherapy (ORR 74.4% (66.2–81.6) versus 51.9% (43.0–60.7), 95% CI 11.2–33.7, *p* = 0.00006). The complete response rate (CR) was also beneficial in the triple combination with pembrolizumab (11.3% vs. 3.1%), as well as the disease control rate (DCR, 95% CI 96.2% (91.4–98.8) versus 89.3 (82.7–94.0) [6]. This clear significant increase in ORR of adding pembrolizumab to the standard-of-care first-line combination of trastuzumab plus chemotherapy led to the approval of this combination for patients with Her2-positive metastatic EGC in the USA and is expected in Europe next year.

For patients with Her2-overexpressing EGC in advanced or metastatic disease stage, the phase II INTEGA trial assesses a superior effect on OS by the chemotherapy-free combination of Her2-blockade (trastuzumab) plus immune checkpoint inhibition (nivolumab + ipilimumab) in comparison with nivolumab plus the standard first-line regimen (trastuzumab + FOLFOX chemotherapy) [40]. First results of the efficacy analysis demonstrate an increased efficacy of the combination of trastuzumab, nivolumab and FOLFOX compared with the TOGA regimen. Furthermore, combining Her2-blockade, immunotherapy and chemotherapy prolonged the OSR compared with the chemotherapy-free study arm independently of PD-L1 CPS expression (all patients: 70% vs. 57%, *p* = 0.034). This combination further improves PFS (all patients: 10.7 vs. 3.2 mths) [30]. The additional cytotoxic effect of chemotherapy seems to intensify the antitumor immune response, but further investigations will be needed to verify the advantage in survival of the triple combination compared with a chemotherapy-free therapy regimen.

A new therapy approach in Her2-targeted therapy of advanced EGC is the use of the antibody-drug conjugate (ADC) T-DXd in patients with Her2-overexpressing locally advanced, unresectable or metastatic tumors. T-DXd consists of an anti-Her2 antibody, a tetrapeptide-based linker and a membrane-permeable topoisomerase I inhibitor payload. The internalization in the Her2-positive tumor cell is aimed to reduce systemic cytotoxicity and to enhance tolerability of this ADC. Promising results of a dose expansion phase 1 trial demonstrated the antitumor activity of T-DXd monotherapy (ORR 43.2%) in 44 patients who received several therapy lines including trastuzumab [8].

The DESTINY-GASTRIC 03 phase Ib/II trial investigates the efficacy of T-DXd in several combinations including immunotherapy. In the dose escalation part (part 1), patients with prior trastuzumab therapy receive either T-DXd combined with 5-FU/capecitabine/the PD-1 inhibitor durvalumab/5-FU or capecitabine plus oxaliplatin/5-FU or capecitabine plus durvalumab. In the dose expansion part (part 2), therapy-naive metastatic patients are stratified by HER2 status and randomized in four study arms of T-DXd, trastuzumab plus 5-FU/capecitabine plus oxaliplatin/cisplatin, T-DXd plus 5-FU/capecitabine +/− oxaliplatin or T-DXd plus 5-FU or capecitabine plus durvalumab. Primary endpoints comprise safety, dose finding (part 1) and ORR (part 2) [31]. As currently presented at ASCO GI 2022, the part 1 results suggest the tolerability and feasibility of the recommended phase 2 doses for T-DXd plus 5-FU and T-DXd plus capecitabine. The ORR results of both arms are promising [9]. Recruitment of patients is ongoing.

A further Her2-targeted antibody is the specific Fc-domain optimized anti-Her2 antibody margetuximab, which further activates the innate and adaptive immune system by antibody-dependent cellular cytotoxicity (ADCC) and anti-Her2-targeted T-cell response. In vitro, margetuximab enhanced the tumor cell-specific PD-L1 expression, which additionally induces an antitumor activity by the PD-1/PD-L1/2 pathway. The global phase II/III MAHOGANY trial investigates combination therapy of margetuximab with the anti-PD-1 antibodies retifanlimab and tebotelimab with/without chemotherapy (XELOX/mFOLFOX) in 860 patients [11]. The combination therapy of anti-Her2 blockade with immune checkpoint inhibition and cytotoxic chemotherapy is expected to enhance antitumoral immunity. The first results of the safety analysis of 43 PD-L1-positive (CPS ≥ 1), non-microsatellite instability high patients treated with the chemotherapy-free combination of margetuximab plus retifanlimab (cohort A) were presented at ESMO 2021. A tumor shrinkage of 85.7% (30/35 patients) with at least one post-baseline target lesion measurement was reported. Furthermore, the combination was well tolerable, with the most common treatment-related adverse events being infusion-related reaction (18.6%, 8/43 patients), diarrhea and fatigue (each 14%, 6/43 patients) [41]. After this safety analysis, a randomized study design follows, with combination of margetuximab, PD-1 inhibition with/without chemotherapy compared to the standard therapy of trastuzumab plus chemotherapy.

In Her2-positive patients, immune checkpoint inhibition also prolongs OS with approved access in the USA and awaited approval in Europe in 2023.

However, one future challenge remains to identify and address trastuzumab-resistant patients with loss of Her2 expression. Several studies have analyzed changes in Her2 status after progression of first-line Her2-directed therapy to clarify the lack of survival advantage in the second-line setting of anti-Her2 therapy in Her2-positive advanced GC patients: a multicenter observational study re-evaluated the Her2 status in patients with advanced or recurrent GC refractory to trastuzumab in order to identify possible biomarkers for loss of response to Her2-targeted first-line therapy with trastuzumab [42]. The Her2 status from biopsy samples of patients showing resistance to trastuzumab was evaluated before and after development of trastuzumab resistance. Loss of Her2 expression was detected in 60.6% of patients with refractory disease after first-line trastuzumab (20/33). Immunohistochemical Her2 overexpression was clearly decreased after trastuzumab treatment. These data indicate that Her2 status should be re-evaluated especially in Her2-positive patients who progressed after trastuzumab first-line therapy. The lack of effect of Her2-directed agents is further analyzed using margetuximab in combination with pembrolizumab in 60 Her2-positive advanced EGA patients in second-line settings post trastuzumab progression and the results recently presented at ASCO GI 2022. By ctDNA-analysis, 61% *ERBB2* amplification was detected and predicted response to combination therapy of margetuximab plus pembrolizumab (24% vs. 0% (*p* = 0.0655), especially in PD-L1-positive patients. In the subgroup of ctDNA- and PD-L1-positive GC patients, ORR was 57% and DCR was 86% [43]. Further research is needed to filter responders from non-responders to Her2-directed therapy after trastuzumab progression and to clarify the role of re-evaluation of the Her2 status (*ERBB2* amplification) beyond progression as a possible predictive biomarker for response to Her2-targeted therapy regimes in second-line post trastuzumab settings.

**Figure 1:**
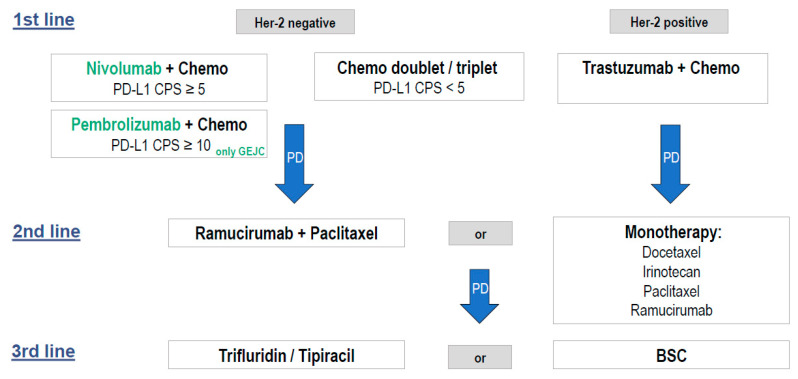
New therapeutic strategies in advanced esophagogastric adenocarcinoma (Europe). Chemo = chemotherapy, PD-L1 CPS = programmed cell death protein 1 (combined positivity score), BSC = best supportive care.

A possible combination of the new approved therapeutic strategies of Her2-negative and Her2-positive advanced esophagogastric adenocarcinoma treatment in Europe is provided in Figure 1.

## 7. Molecular Biomarkers in Advanced Gastric Cancer

Her2-overexpression is an established predictive biomarker in advanced EGC with implementation in therapy guidelines. Further biomarkers are under investigation to identify molecular characteristics of subgroups of patients and to find an individual antitumor treatment in addition to chemotherapy:

### 7.1. PD-L1 CPS

The PD-L1 CPS score has increasingly been developed as a predictive marker for response to immunotherapy in adenocarcinoma of the stomach and GEJ and is being regularly used as a stratification marker in trials. PD-L1 CPS comprises the total of positive stained tumor cells and positive stained mononuclear immune cells including lymphocytes, macrophages, and dendritic cells (with simultaneous consideration of membranous and cytoplasmic staining) divided by the total population of tumor cells [44]. In gastric cancer, PD-L1 CPS ≥ 1 generally defines PD-L1-positive tumors. The PD-L1 CPS score of choice for prediction of efficacy of immunotherapy is still under investigation in clinical trials (see above) and varies between CPS ≥ 1/CPS ≥ 5 and CPS ≥ 10. In pembrolizumab trials, CPS ≥ 10 showed an effective discrimination of response to therapy, while in nivolumab trials, a cut-off of CPS 5 was used for primary endpoints OS and PFS. In general, response to immunotherapy with prolongation of OS is demonstrated to be dependent on the amount of PD-L1 CPS positivity: the higher the CPS score, the higher the benefit in OS. This is seen in the CHECKMATE-649 trial with nivolumab (higher OS benefit with CPS ≥ 5 versus CPS ≥ 1) and in the KEYNOTE-062/061 trials with pembrolizumab (higher OS benefit with CPS ≥ 10 versus CPS ≥ 1). A recent comprehensive analysis of selected clinical trials (KEYNOTE-059, KEYNOTE-061, KEYNOTE-062) with administration of pembrolizumab in patients with CPS ≥ 10 further confirms this finding [45].

Nonetheless, there remain some unanswered questions on how to handle the PD-L1 CPS score as a reliable tool to differentiate between responders and non-responders to immune checkpoint inhibitor therapy of GC in the future.

The question of a reliable cut-off value of the PD-L1 CPS score as a consistent marker to predict benefit from immune checkpoint inhibition needs to be clarified. As mentioned above, recent phase II and III trials indicate a survival benefit of immune checkpoint therapy, especially in patients with high CPS-scores (CPS ≥ 5). The assumed lack of benefit of the subgroup of patients with low CPS scores (CPS ≤ 5, ≤1) was investigated in randomized phase III trials (CHECKMATE-649, KEYNOTE-062 and KEYNOTE-590) that compared the addition of immune checkpoint inhibitors with chemotherapy using a Kaplan–Meier subtraction approach. The comparison analysis confirmed that the patients with low PD-L1 CPS scores (subgroups of CPS 1-9 and CPS 1-4) investigated in these trials did not show significant benefit from the addition of immune checkpoint inhibitors compared with standard chemotherapy in advanced EGC [46]. We personally recommend to further classify the subgroup of low PD-L1 CPS patients by specific analyses and to reflect the rational use of immune checkpoint inhibitors in patients with low CPS scores.

The magnitude and consistency of PD-L1 as a predictive marker is the object of investigation of a current systematic review and meta-analysis of 14 phase III trials in advanced EGC. The primary results were presented at ASCO GI 2022 and identified PD-L1 CPS as the second strongest predictive biomarker for survival benefit from immune checkpoint inhibition compared to standard-of-care after MSI [47]. This study also confirms further findings of gender differences in response to immune checkpoint therapy as already demonstrated, for example, in KEYNOTE-590 with lower benefit from pembrolizumab plus chemotherapy vs. placebo for women compared to men (HR = 0.89 (0.59–1.35) vs. HR = 0.70 (0.58–0.84), 95% CI) [19]. We strongly support the clarification of the sex-specific differences in response to immunotherapy in future immune checkpoint inhibitor trials and to work out the possible role of sex as a predictive marker for immune checkpoint inhibitors.

### 7.2. MSI

The MSI group of gastric cancer patients resembles one of the four molecular subgroups proposed by the TCGA next to chromosomally instable tumors, Epstein–Barr virus-infected tumors and genomically stable tumors [48]. There is growing evidence that the MSI status in advanced gastric cancer is positively correlated with response to immunotherapy in advanced gastric cancer. Microsatellite instable tumors exhibit an intrinsic mutational burden and supply tumor neoantigens, increasing the sensitivity to immunotherapy [49]. A meta-analysis compared randomized trials which investigated treatment with or without a PD-1 inhibitor for advanced gastric cancer including provided outcomes in reference to MSI status (KEYNOTE-062, KEYNOTE-061, CHECKMATE-649, JAVELIN Gastric 100); the data were selectively examined according to the role of MSI in response to immunotherapy. In total, 2545 patients with evaluable MSI status were included with 123 MSI-high GC patients (4.8%). In MSI-high tumors, the HR for OS benefit by anti-PD-1 therapy was 0.34 (95% CI 0.21–0.54) compared to 0.85 (95% CI 0.71–1.00) for MSS (microsatellite stable) tumors. The HR for the PFS in MSI-high tumors was 0.57 (95% CI 0.33–0.97, *p* = 0.04) and the odds ratio for response was 1.76 (95%CI 1.10–2.83, *p* = 0.02) [50]. This analysis strengthens the hypothesis of MSI-high GC patients being a highly immunosensitive population that is particularly responsive to immunotherapy.

Further studies are needed to clarify whether patients with high levels of microsatellite instability or deficient mismatch repair (dMMR) in particular qualify for single/combination immunotherapy. There are subgroup analyses indicating no survival benefit for patients with MSI-high EGC treated with chemotherapy in the perioperative setting compared with surgery (meta-analysis of MAGIC, CLASSIC, ARTIST, ITACA-S trial) [51] As presented at ESMO 2021, results of the DANTE trial show benefit for MSI-high patients treated with a combination of immune checkpoint inhibitors and chemotherapy, with rates of pathological complete response or subtotal regression (TRG1a/b) being 80% (8/10) after FLOT plus atezolizumab vs. 59% (7/12) after FLOT monotherapy [52]. Furthermore, the role of neoadjuvant nivolumab plus ipilimumab and adjuvant nivolumab with localized MSI-high/dMMR EGC was investigated in the GERCOR NEONIPIGA phase II trial with the primary endpoint of pathological complete response rate (pCRR). First results presented at ASCO GI 2022 showed neoadjuvant immune checkpoint inhibition as a feasible therapy option. The pCRR was 59% (17/29 patients) and 94% of patients were free of events after 12 months of follow up [53]. These results raise the question whether immunotherapy postpones or even replaces surgery in patients with MSI-high/dMMR tumors. We recommend that testing the MSI status of EGC patients should be routinely performed at the time of diagnosis prior to treatment. With future larger studies, we might really implement immunotherapy in perioperative settings, especially for patients with MSI-high/dMMR tumors.

### 7.3. EBV

Latent Epstein–Barr Virus (EBV) infection is predominant in nearly 9% of gastric adenocarcinoma patients [54]. GC patients with EBV-positive tumors exhibit a higher amount of immune checkpoint genes such as PD-1 or CTLA-4 and a higher level of lymphocytic infiltration compared to MSS (microsatellite stable) tumors [44]. The role of the EBV-positive status of gastric tumors as a possible predictive marker of the response to immunotherapy is investigated in several clinical trials. In an Asian study with 300 gastric cancer patients, the PD-L1 CPS score ≥ 1 (59.3%, 178 patients) was significantly associated with MSI-high tumors and a positive EBV status. These results indicate EBV-positive GC patients to particularly benefit from immunotherapy [55].

## 8. Conclusions

Immunotherapy is a rapidly developing field of research in gastric cancer treatment. Approvals of immune checkpoint inhibitors for advanced EGC patients enhance the current treatment options and constitute a feasible, personalized therapy option. Ongoing phase II and III trials enable patients access to immunotherapy in every line of therapy. The combination therapy of nivolumab plus chemotherapy achieved a clinically meaningful OS benefit in the first-line setting in all advanced esophageal and gastric adenocarcinoma patients with approval in Europe (CPS ≥ 5), the USA, Taiwan and other countries. For patients with esophageal cancer and Siewert-1 GEJC, the combination of pembrolizumab plus chemotherapy is approved in Europe (CPS ≥ 10) and the USA. In the third-line setting, nivolumab prolonged OS compared with placebo and was approved in Japan. Furthermore, pembrolizumab prolonged duration of response significantly, resulting in approval for patients with PD-L1 CPS ≥ 1 tumors in the USA. For patients with Her2-overexpressing tumors, combination of trastuzumab, pembrolizumab and chemotherapy shows superior effect on response and is approved as a first-line therapy option in the USA. In the progression of immunotherapy in adenocarcinomas of the stomach/gastroesophageal junction, it remains necessary to further identify and subdivide subgroups and sex-specific differences of patients who particularly seem to benefit from response to immunotherapy by possible predictive biomarkers (PD-L1 CPS, MSI, EBV) within the upcoming years.
